# Comparison of Different Blood Lactate Threshold Concepts for Constant Load Performance Prediction in Spinal Cord Injured Handcyclists

**DOI:** 10.3389/fphys.2019.01054

**Published:** 2019-09-27

**Authors:** Carolin Stangier, Thomas Abel, Sebastian Zeller, Oliver Jan Quittmann, Claudio Perret, Heiko K. Strüder

**Affiliations:** ^1^Institute of Movement and Neurosciences, German Sport University Cologne, Cologne, Germany; ^2^European Research Group in Disability Sport, Cologne, Germany; ^3^Institute of Sports Medicine, Swiss Paraplegic Centre, Nottwil, Switzerland

**Keywords:** endurance capacity, endurance exercise, spinal cord injury, lactate minimum test, graded exercise test

## Abstract

**Background:**

Endurance capacity is one of the main performance determinants in handcycling. There are two exercise test procedures primarily applied to determine endurance capacity, to verify training adaptations and predict race performance. This study aims to evaluate the agreement of these applied concepts in handcycling.

**Methods:**

In a repeated measures cross-over design, 11 highly trained male spinal cord injured (Th12 to L1) handcyclists (age: 40 ± 9 years, height: 183 ± 8 cm, body mass: 73.2 ± 8.5 kg) performed a graded exercise test (GXT) and a lactate minimum test (LMT) to determine lactate threshold at 4 mmol L^–1^ (LT_4 mmol L_−1) and lactate minimum (LM)_,_ respectively. The agreement of both lactate thresholds concepts for constant load performance prediction (change of ≤ 1 mmol L^–1^ during the last 20 min) was evaluated within constant load tests (CLT; 30 min) at a power output (PO) corresponding to LT_4 mmol L__–__1_ and LM. Oxygen uptake ($\dot{\text{V}}\,{\text{O}}_{2}$), respiratory exchange ratio (RER), heart rate (HR) and blood lactate (La) were measured during all tests.

**Results:**

Power output at the corresponding thresholds (LT_4 mmol L_−_1_: 149 ± 34 W vs. LM: 137 ± 18 W) revealed no significant difference (*p* = 0.06). During the CLT at LT_4 *mmol*__⋅__*L*_−_1_ and LM, $\dot{\text{V}}\,{\text{O}}_{2}$, and RPE were not significantly different. However, LA, RER, and HR were significantly higher (*p* ≤ 0.02) during CLT at LT_4 mmol L_−^1^. Bland–Altman plots indicate a wide range of dispersion for all parameters between both lactate threshold concepts. Evaluations of LT_4 mmol L_−_1_ and LM did not meet the criteria for constant load performance within the CLT for 33 and 17% of the athletes, respectively.

**Discussion:**

Both exercise tests and the corresponding lactate threshold concept revealed appropriate estimates to predict a steady state performance for the majority of participants. However, as PO determination at LT_4 mmol L_−_1_ and LM exceeds the criteria for constant load performance (increase of ≥ 1 mmol L^–1^) for 33 and 17% respectively the current results indicate the common criteria for constant load performance (change of ± 1 mmol L^–1^) might not be sufficiently precise for elite athletes in handcycling. Consequently, exercise test results of elite athletes should be analyzed individually and verified by means of several CLT.

## Introduction

The first world championships in handcycling were staged in 1999 and the debut at Paralympic Games was 2004 in Athens. Forty-four male and 25 female athletes from 25 countries competed in the previous Paralympic Games 2016 in Rio. Based on the results handcycling appears to be dominated by European athletes. Between 2004 and 2016, 87% of all medals have been won by athletes from Europe. As race distances range between 20 and 30 km for time trials and between 50 and 80 km for road races with mass starts, dependent on gender and classification, endurance capacity is one of the main performance determinants in handcycling. To date there are two exercise test procedures primarily applied to determine endurance capacity in handcycling; the lactate minimum test (LMT) (Perret et al., 2012) and the graded exercise test (GXT) to exhaustion (Zeller et al., 2015). The obtained test results allow the analysis of current endurance capacity to verify training adaptations and are routinely used as a time-efficient alternative to predict maximum lactate steady state (MLSS) (Jones and Doust, 1998; Billat et al., 2003; Faude et al., 2009; Adam et al., 2015). MLSS is defined as the maximum exercise intensity at which lactate production and elimination are equilibrated and represents a more robust parameter for performance prediction and training description than $\dot{\text{V}}\,{\text{O}}_{2}$ max in able-bodied athletes (Coyle et al., 1988; Jones and Rikli, 2000; Fahs et al., 2014). The LMT protocol involves a ramp test until volitional exhaustion to induce a hyperlactemia. This is followed by a recovery phase allowing an equilibration of lactate concentration between muscle and blood. The subsequent incremental exercise test is either controlled by heart rate (Strupler et al., 2009; Perret et al., 2012), speed (Macintosh et al., 2002) or performance (Johnson et al., 2009; Knoepfli-Lenzin and Boutellier, 2011) response that evokes a u-shape of blood lactate concentration. Lactate minimum (LM) – estimating MLSS – is determined by taking the performance at the nadir of blood lactate concentration during the incremental test. The GXT protocol consists of graded increases in exercise intensity from a very low relative or absolute exercise intensity until exhaustion. MLSS is estimated for example at a fixed blood lactate concentrations of 4 mmol L^–1^ (LT_4 mmol L__–__1_; Heck et al., 1985).

There is considerable evidence for the validity of both the GXT and LMT in prediction of the MLSS in able-bodied individuals (Jones and Doust, 1998; Beneke, 2003b; Billat et al., 2003; Faude et al., 2009; Adam et al., 2015). However, with increasing endurance performance capacity the LMT might result in an underestimation of power output (PO) at LM compared to MLSS (Heck et al., 1991). As endurance training induced structural and metabolic adaptations affect blood lactate kinetic during exercise, the accuracy of LM could be impaired (Hood and Saleem, 2007). A lower maximal blood lactate concentration after the first part (ramp test) combined with possibly higher blood lactate elimination at the beginning of the second part (step test) could lead to a rather low blood lactate concentration around the minimum in highly trained athletes. Consequently, are a premature net-blood lactate accumulation and a left displacement of the lactate curve can occur resulting in an underestimated PO at LM (Knoepfli-Lenzin and Boutellier, 2011). Similar but inverse results are verified for the fixed 4 mmol L^–1^ lactate threshold which can overestimate the MLSS in highly trained endurance athletes (Faude et al., 2009). These limitations have to be considered when LM or LT_4 mmol L__–__1_ are applied for MLSS prediction in able-bodied athletes.

In comparison to bicycling, propulsion in handcycling is produced with the upper body involving smaller muscle groups that have to accelerate a higher mass (three wheeled handbike vs. bicycle). Moreover, biomechanical analyses revealed a considerably lower mechanical efficiency for arm-crank ergometry like handcycling (15 to 18%) than for cycle ergometry (23 to 25%) (Glaser, 1985; Dallmeijer et al., 2004; Abel et al., 2006). Combined with the spinal cord injury (SCI) related physiological limiting factors (e.g., venous blood pooling) (Theisen et al., 2001a) the sport-specific demands for athletes in handcycling results in a different physiological pattern compared to traditional bicycling for able-bodied athletes. Previous studies investigating SCI individuals indicate that absolute $\dot{\text{V}}\,{\text{O}}_{2}$ at the lactate threshold do not differ significantly between able-bodied individuals and individuals with high and low level paraplegia during arm crank ergometry (Flandrois et al., 1986; Bhambhani, 2002). As paraplegia leads to lower $\dot{\text{V}}\,{\text{O}}_{2}$ peak values the lactate threshold occurs at a higher percentage of $\dot{\text{V}}\,{\text{O}}_{2}$ peak. This relation increases with the height of lesion level (Bhambhani, 2002). Data from Perret et al., 2012 showed a close relationship between heart rate responses at LM and at MLSS in wheelchair racing but noted that the heart rate at MLSS can be assumed to be 8–9 bpm above the one at LM (Perret et al., 2012).

Although there is evidence for inactive skeletal muscle playing a role in the lactate metabolism of able-bodied individuals, paralyzed athletes have a similar lactate elimination during arm-cranking exercise as able-bodied athletes (Leicht and Perret, 2008). Schantz et al. (1997) noted a general trend toward a higher proportion of type-I fibers and lower proportion of type-IIx fibers in the anterior portion of the paralyzed deltoid muscle. Nevertheless, during late exercise and early recovery elimination rates were higher for able-bodied athletes (Leicht and Perret, 2008). As slowed elimination rates in the early stage of recovery for paralyzed athletes might affect accuracy for LM threshold determination that may result in an underestimation of MLSS. Thus, lactate concentration would increase earlier during the second test part (step test) due to the delayed lactate elimination representing a minor recovery.

However, there is no study available comparing the validity of different exercise testing procedures in handcycling. Due to several methodological differences (testing procedure, ergometer, performance level) in the single studies and the wide range of lesion levels of the examined athletes a comparison between different studies appears ineffectual. Since the homogeneity in the performance and endurance capacity of paraplegic athletes is considerably high, even within the same lesion level (Molik et al., 2010; Morgulec-Adamowicz et al., 2011), the requirement for a standardized exercise testing procedure is vital to allow international and interindividual comparisons of athletes in handcycling. Therefore the definition of an international standard for exercise testing would be desirable. For these reasons the purpose of this study was to investigate the agreement of the LMT and GXT for constant load performance prediction in highly trained SCI athletes. On the basis of the current scientific knowledge it is hypothesized that both testing procedures are appropriate to predict constant load performance but depending on athlete’s fitness level the GXT is more likely to overestimate and the LMT to underestimate the constant load performance.

## Materials and Methods

### Participants

Twelve male elite athletes competing on an international level in handcycling including Paralympic medal winners (2012, 2016) were recruited for the study. All athletes were categorized in classes H3 and H4 of the Union Cycliste Internationale (UCI) classification system. As one lesion level might already involve a wide range of performance levels, homogeneity of participants in the current study is ensured by similar UCI-classifications (Molik et al., 2010; Morgulec-Adamowicz et al., 2011). Moreover, all athletes showed a motor complete SCI (AIS B). Participants’ individual anthropometric characteristics, disability and UCI classification are presented in Table 1. Athletes with spinal lesion levels between Th1-5 are also likely to achieve lower than anticipated maximal heart rate values, while those with lesions below that level will have normal responses (Hopman et al., 1993b; Hopman, 1994; Theisen, 2012). For this reason, only athletes with a lesion level below Th4/5 were included in this study and peak data confirm that none of the athletes have a limitation in HR response (see Table 2); thus indicating a cardiovascular situation comparable to able-bodied individuals. The participants were fully informed of the purposes and risks associated with the study design before providing written, informed consent. The study conformed to current local guidelines and the Declaration of Helsinki and was approved by the German Sport University Ethics Advisory Committee.

**TABLE 1:** Athletes’ anthropometric characteristics, disability, and UCI classification.

	**Age**	**Body mass**	**Body fat**	**Lesion level**
	**yrs**	**kg**	**%**	
Mean	40.1	73.2	13.7	
*SD*	9.3	8.5	1.7	
Range	31; 58	61.3; 84.0	10.0; 16.6	L1; Th4/5

**TABLE 2:** Peak responses and correlation between the different exercise test procedures.

				**Correlation**	
		**LMT**		**GXT –**	
	**GXT**	**(ramp test)**		**LMT_ramp**	
			***p*-value**	***r***	***p***
$\dot{\text{V}}\,{\text{O}}_{2}$ (ml ⋅ min^–1^ ⋅ kg^–1^)	40.5 ± 6.2	40.7 ± 6.7	0.513	0.827^∗^	0.002
RER	1.03 ± 0.05	1.18 ± 0.14	0.006	0.414^∗^	0.206
HR (bpm)	183 ± 12	174 ± 10	0.027	0.735	0.006
BLa (mmol⋅L^–1^)	11.5 ± 2.8	8.2 ± 1.5	0.001	0.531	0.075
RPE	19.9 ± 0.3	19.9 ± 0.1	0.887		
PO (W)	192 ± 29	228 ± 30	0.002	0.866	0.001
Test duration (min)	48.3 ± 7.3	11.4 ± 1.5	0.0001		

**n* = 12; GXT, graded exercise test; LMT, lactate minimum test; *r*, correlation coefficient; *p*, level of significance, ^∗^spearman correlation.*

### General Procedures

To prevent possible bias related to circadian rhythms, all exercise testing were scheduled at the same time of day. Participants were advised not to take part in vigorous exercise during the 2 days before the tests to prevent glycogen depletion. In order to avoid any disruption to the athletes’ training routine all measurements were conducted on two consecutive days in the laboratory. Athletes were instructed to arrive in a rested, carbohydrate-loaded and fully hydrated state on each testing day (Jeacocke and Burke, 2010). A familiarization session was not necessary because all exercise tests were performed on subjects’ individual handcycles mounted on a validated cycle ergotrainer (Cyclus2, RBM Electronics; Leipzig, Germany; Reiser et al., 2000). The cranks were set for the synchronous mode of cranking preferred by all subjects.

Participants initially underwent a medical healthcare check involving a resting electro cardiogram, a hemogram and a questionnaire to exclude any health risk. Prior to the exercise tests, body height and mass were measured and recorded. Percentage of body fat was calculated from skinfold thickness measurements (Harpenden Skinfold Calipers, Baty International, West Sussex, United Kingdom) at 10 sites (Parizkova, 1961; Perret and Abel, 2016). Subsequent to the medical and anthropometric measurements, athletes randomly completed the GXT or the lactate minimum test (LMT) and then continued with the corresponding constant load test (30 min). Both tests were separated by at least 2 h to provide enough time for recovery represented by the return to resting state of blood lactate concentration (Monedero and Donne, 2000; Leicht and Perret, 2008). The remaining exercise test and the corresponding constant load test (30 min) were completed on the second testing day in the same sequence as the day before.

During the exercise tests, intensity increased step or ramp wise until subjects were exhausted or they failed to maintain the pedaling cadence (50 rpm) or speed (Zeller et al., 2015). Exhaustion was considered with the attainment of at least two of the following criteria: a plateau in oxygen uptake ($\dot{\text{V}}\,{\text{O}}_{2}$) despite increasing work rate; high levels of blood lactate concentration (BLa; 8–10 mmol L^–1^); a respiratory exchange ratio (RER) above 1.10, and/or a heart rate (HR) of ± 10 bpm of age-predicted maximum (220-age) (Hollmann et al., 2012).

### Instrumentation

During each test, breath by breath measurements of $\dot{\text{V}}\,{\text{O}}_{2}$ and carbon dioxide output ($\dot{\text{V}}\,{\text{CO}}_{2}$) were recorded through a spirograph (ZAN 600, nSpire Health, Inc., Longmont, CO, United States). The highest values within the last 30 s of each speed level were used for analysis. Calibration and ambient air measurement were conducted before each testing session using a precision 1-L syringe and calibration gas (15.8% O_2_, 5% CO_2_ in N; Praxair, Düsseldorf, Germany). HR was continuously measured by a HR monitor (S810i; POLAR, Büttelborn, Germany). To determine BLa, arterialized blood samples (20 μl) were extracted from the hyperemic earlobe after each step, immediately placed in a hemolyzing solution, and analyzed in our laboratory (BIOSEN C-line; EKF, London, United Kingdom). Rating of perceived exertion (RPE) was evaluated using the Borg-scale 6–20 directly after each step (Borg et al., 1987).

### Exercise Testing

#### Graded Exercise Test (GXT)

Subsequent to a low-intensity self-paced warm-up phase (10 min), the maximal intensity GXT started with an initial workload of 20 W which was increased every 5 min by 20 W until exhaustion (Zeller et al., 2015). During the last 30 s of every step, capillary blood samples were taken to measure BLa and RPE was noted. In addition to peak physiological response measurements, this test enables the identification of the PO corresponding to a fixed blood lactate concentration of 4 mmol L^–1^ by using linear interpolation methods (Heck et al., 1985) and was used for the related CLT. This exercise intensity has been shown to be the best metabolic predictor of simulated, laboratory-based handcycle race performance (Abel et al., 2003).

#### Lactate Minimum Test (LMT)

Prior to the test athletes completed a low-intensity self-paced warm-up phase (10 min). To induce hyperlactemia before commencing a standard incremental exercise test, athletes performed a ramp protocol. The initial workload of 20 W increased every 60 s by 20 W until exhaustion. After a passive recovery of 2 min the incremental test started with an individualized workload corresponding to 45% of maximal PO within the ramp test. This exercise intensity increased by 15 W every 5 min until exhaustion (adapted from Perret and Vrana, 2014). During the last 30 s of every step, capillary blood samples were taken to measure BLa and a value for RPE was noted. The LM was defined as the PO at which a curve fitted to the “U-shaped” blood lactate data derived from the incremental test reached a nadir. This point was supposed to represent a point of equilibrium between lactate production and removal (Jones and Doust, 1998). Performance was chosen as intensity control due to the unit of the ergometer (Cyclus2, RBM Electronics; Leipzig, Germany) and since heart rate response might be affected by SCI. In contrast to methods utilizing blood lactate concentrations, the LM is apparently unaffected by conditions of glycogen depletion (Tegtbur et al., 1993). PO corresponding to the LM represented the workload for the related CLT.

#### Constant Load Test (CLT)

To verify the prediction of constant load performance for both exercise test procedures and lactate threshold concepts athletes completed a constant load test each at freely chosen cadence. After a standardized warm-up of 3 min at 60%, 3 min at 80%, and 4 min at 100% of the particular threshold PO, athletes completed 30 min of constant PO corresponding to the power at LT_4 mmol L__–__1_ determined within the GXT on one testing day and at the PO corresponding to LM from the LMT on the other testing day (adapted from Perret and Vrana, 2014). Both CLT included continuous measurements of HR, $\dot{\text{V}}\,{\text{O}}_{2}$, $\dot{\text{V}}\,{\text{CO}}_{2}$, and cadence. Capillary blood samples and RPE values were obtained at 5 min intervals. Achievement of constant load performance was confirmed by a change of ≤ 1 mmol L^–1^ in BLa during the last 20 min.

### Data Analysis

Data are presented as mean ± standard deviation (SD) and were analyzed using SPSS Statistics 24 (IBM Deutschland GmbH, Ehningen, Germany). A Kolmogorov–Smirnov test was used to confirm normal dispersion and a Mauchley test of sphericity to verify homogeneity of variance. Peak physiological responses and performance outputs in GXT and LMT (ramp test, step test) were compared within a one-way ANOVA or Friedman ANOVA ($\dot{\text{V}}\,{\text{O}}_{2}$ peak, RERpeak). A two-way ANOVA (parameter × time course) or Friedman’s ANOVA was used to compare submaximal responses (VO_2_, HR, RER, La, RPE) during the constant load test (at 5, 10, 15, 20, 25, 30 min) at the two predicted POs (4 mmol L^–1^ and lactate minimum). PO at the defined lactate thresholds (LT_4 mmol L__–__1_ and LM) were compared within a paired *t*-test. Additionally, Bland–Altman plots were produced to illustrate the comparison of peak responses between both exercise tests and for submaximal responses during the CLT at lactate thresholds. To examine the relationship between the peak values and between the physiological responses during the CLT, the Pearson’s product-moment or Spearman correlation coefficient test was computed for both exercise tests. Statistical significance was accepted at a level of *p* ≤ 0.05.

## Results

### Exercise Test Procedures

As presented in Table 2 all athletes fulfilled the test criteria for exhaustion.

Except for $\dot{\text{V}}\,{\text{O}}_{2}$_peak_ and RPE all parameters showed significant differences between the GXT and the LMT. While the LMT revealed the highest values for RER and PO maximum, the peak values for LA and HR were achieved during the GXT (Table 2). A significant correlation between $\dot{\text{V}}\,{\text{O}}_{2}$_peak_ values confirms a comparable exhaustion between both exercise test procedures regarding the pulmonary system (Table 2).

Although the one-way ANOVA revealed significant differences for physiological and performance responses between both testing procedures there are also strong significant correlations for peak HR, La and PO values between the GXT and LMT (Table 2).

**FIGURE 1:**
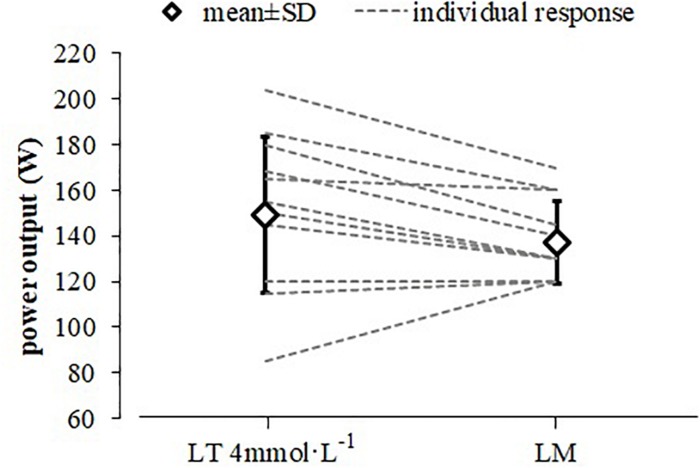
Mean and individual power output responses at the LT_4 mmol L_^–1^ and lactate minimum (LM) to predict constant load performance. Only 11 lines are visible between both data points for the individual responses as two participants had identical power outputs for both threshold concepts (LT_4 mmol L_^–1^ and LM).

**FIGURE 2:**
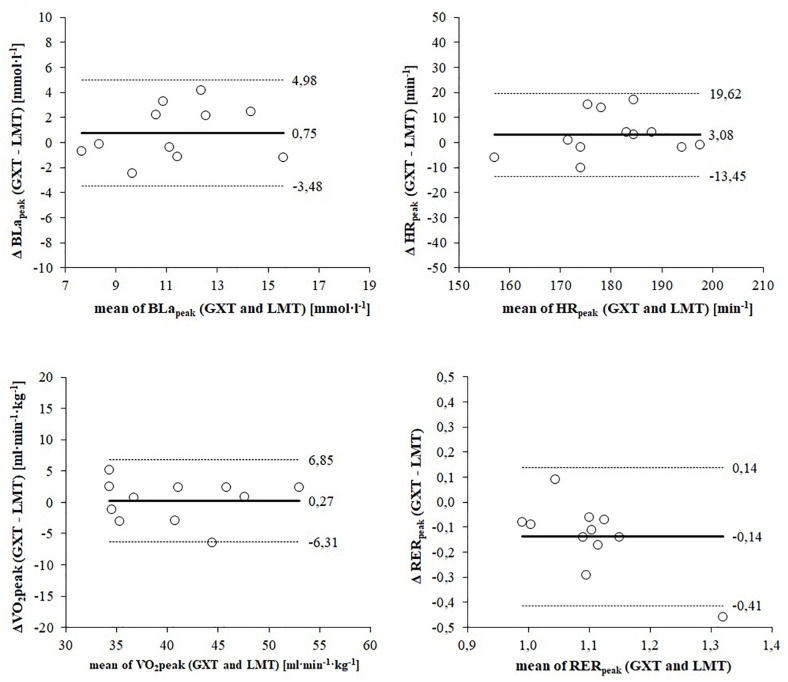
Bland–Altman plots displaying agreement of peak responses between the graded exercise test (GXT) and lactate minimum test (LMT). The differences between measures (y-axis) are plotted as a function of the two measures (x-axis) in the unit of the physiological parameter. The horizontal continuous line represents the mean difference (bias) and the paired dashed lines represent the limit of agreement (mean ± 2 SD).

Figure 1 shows Bland–Altman plots depicting the agreement of physiological peak responses between GXT and LMT. The mean difference (bias) of BLa_peak_ was 0.75 mmol L^–1^ higher for the GXT and showed a high dispersion that is consistent over the range of values for both exercise tests (Figure 2). The mean difference (bias) of HR_peak_ was 3 bpm higher for the GXT and limits of agreement (LoA) indicated a wide range of dispersion (Figure 2). Mean differences of peak responses for VO_s_ showed a good agreement between GXT and LMT (bias: 0.27 ml min kg^–1^). However, a dispersion of 6.3 to 6.9 ml min kg^–1^ is still considerably high when determining VO_2__peak_ (Figure 2). RER_peak_ was the only parameter showing a lower mean difference (bias) of 0.14 for the GXT (Figure 2).

### Constant Load Performance Prediction

The determined PO (149 ± 34 W) at the LT_4 mmol L_−1 represents 77.4 ± 10.9% of the maximumPO in the related GXT. The determined PO (137 ± 18 W) at the LM represents 60.1 ± 2.2% of maximum PO in the ramp test of the related LMT (Figure 2). A paired *t*-test revealed no significant difference between these PO with a medium effect size (*d* = 0.60) and a strong significant correlation (*r* = 0.833, *p* = 0.001).

### Constant Load Test

**FIGURE 3:**
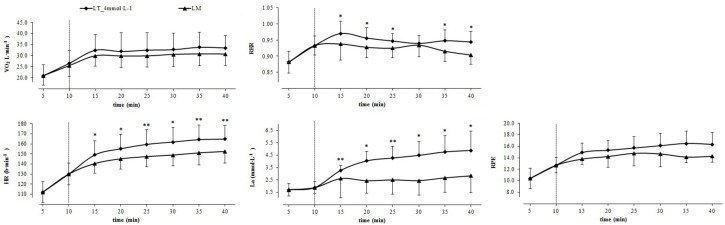
Physiological responses and ratings of perceived exertion during the warm-up (10 min) and the constant load test (30 min) at PO corresponding to 4 mmol L^–1^ threshold (■) and lactate minimum (LM; ▲). Mean ± SD (*n* = 12); ^∗^*p* ≤ 0.05; ^∗∗^*p* ≤ 0.005; the dotted line indicates the end of the 10 min warm-up phase.

$\dot{\text{V}}\,{\text{O}}_{2}$ and RPE responses were not significantly different between the CLT at LT_4 mmol L_−1 and at LM (*p* ≥ 0.06; Figure 3). However, LA (*p* ≤ 0.02) and HR (*p* ≤ 0.02) responses during CLT at LT_4 mmol L__–__1_ were continuously significantly higher (*p* ≤ 0.02) and RER responses were higher for all measurements (*p* ≤ 0.05) except for 20 min (Figure 3). $\dot{\text{V}}\,{\text{O}}_{2}$ responses correlated significantly between both CLT for the full test duration (*r* ≥ 0.631; *p* ≤ 0.037; Table 3).

**TABLE 3:** Sub-maximal responses and correlation between the different lactate threshold concepts during the constant load test.

	**4 mmol L^–1^**		**LM**		**ANOVA**			***Post hoc***		**Correlation**			
	**@10 min**	**@30 min**	**@10 min**	**@30 min**				**@10 min**	**@30 min**	**@10 min**		**@30 min**	
							**Test^∗^**						
					**Test**	**Duration**	**duration**	***p***	***p***	***r***	***p***	***r***	***p***
$\dot{\text{V}}\,{\text{O}}_{2}$(ml⋅min^–1^⋅kg^–1^)	33.7 ± 6.7	33.7 ± 5.6	30.7 ± 5.3	30.5 ± 5.1	0.11	0.22	0.50	0.09	0.02	0.779	0.01	0.760	0.02
RER^∗^	0.95 ± 0.03	0.94 ± 0.04	0.92 ± 0.03	0.89 ± 0.02	0.000			0.02	0.02	0.225	0.53	0.178	0.65
HR (bpm)	164 ± 15	166 ± 14	152 ± 12	154 ± 12	0.01	0.000	0.49	0.01	0.01	0.575	0.06	0.600	0.07
BLa (mmol⋅L^–1^)	4.8 ± 1.3	5.1 ± 1.6	2.7 ± 1.2	2.8 ± 1.4	0.01	0.05	0.17	0.01	0.02	–0.247	0.46	–0.239	0.51
RPE^∗^	16.5 ± 2.2	16.8 ± 2.2	11.1 ± 1.0	14.4 ± 1.3	0.000			0.03	0.06	–0.105	0.80	–0.670	0.89

**n* = 12; LM, lactate minimum; *r*, correlation coefficient; *p*, level of significance; ^∗^Friedman’s ANOVA with a level of significance ≤ 0.008 and spearman correlation.*

Evaluations of PO at LT_4 mmol L__–__1_ and LM did not meet the criteria for constant load performance (increase of ≥ 1 mmol L^–1^ during the last 30 min) within the CLT for 4 and 2 athletes, respectively. Among these 6 athletes both threshold concepts revealed an inappropriate PO for one athlete each resulting in an early test termination of the CLT (test termination at LT_4 mmol L__–__1_ after 20 min, at LM after 15 min).

**FIGURE 4:**
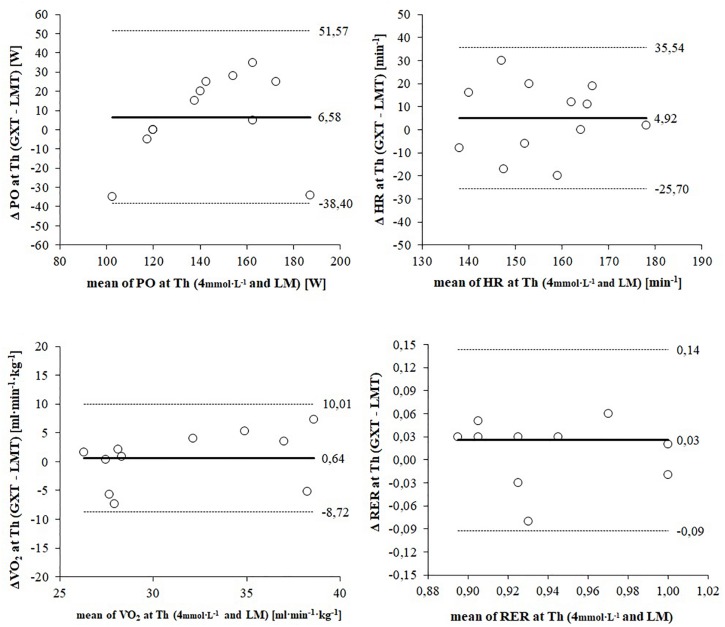
Bland–Altman plots displaying agreement of submaximal responses between the 4 mmol L^–1^ threshold and the lactate minimum (LM). The differences between measures (y-axis) are plotted as a function of the two measures (x-axis) in the unit of the physiological parameter. The horizontal continuous line represents the mean difference (bias) and the paired dashed lines represent the limit of agreement (mean ± 2 SD).

Figure 4 shows Bland–Altman plots depicting the agreement of submaximal responses for PO, HR, VO_2_, and RER between LT_4 mmol L__–__1_ and LM. Mean difference (bias) of PO is 6.58 W higher for the LT_4 mmol L__–__1_ and shows a very high dispersion (Figure 4). According to the dispersion of the data athletes with a lower endurance performance achieve a higher PO at the LM and athletes with a higher endurance performance show a higher PO at the LT_4 mmol L__–__1_. Mean difference (bias) of HR is 5 bpm higher for the LT_4 mmol L__–__1_ and shows a high but consistent dispersion for both lactate threshold concepts (Figure 4). Similar to peak responses the mean difference of VO_2_ shows the best agreement between both lactate threshold concepts (bias: 0.64 ml min kg^–1^) (Figure 4). However, limits of agreement (8.2 to 10.1 ml min kg^–1^) indicate a considerably high dispersion. Mean difference of RER is 0.03 higher for the LT_4 mmol L__–__1_ and shows a wide range of dispersion (LoA: 0.09 to 0.14) (Table 4).

**TABLE 4:** Mean values, mean differences, and limits of agreement (LoA) of peak responses between the graded exercise test (GXT) and lactate minimum test (LMT) and of submaximal responses between the 4 mmol L^–1^ threshold and the lactate minimum (LM).

**Peak parameters**		**GXT**	**LMT**	**Δ**	**LoA**	**LoA**
				**(GXT–LMT)**	−**1.96 SD**	**+ 1.96 SD**
HR_peak_	[min−^1^]	183 ± 12	178 ± 11	3.08	–13.45	19.62
$\dot{\text{V}}$O_2__peak_	[ml⋅min^–1^⋅kg^–1^]	40.5 ± 6.2	41.3 ± 6.3	0.27	–6.31	6.85
RER_peak_		1.03 ± 0.05	1.18 ± 0.14	–0.14	–0.41	0.14
La_peak_	[mmol⋅L^–1^]	11.53 ± 2.77	10.95 ± 2.33	0.75	–3.48	4.98

**Values at threshold**		**4 mmol L^–1^**	**LM**	**Δ**	**LoA**	**LoA**
				**(4 mmol L-^1^–LM)**	**-1.96 SD**	**+1.96 SD**

PO_Th_	[W]	149 ± 34	137 ± 18	6.58	–38.40	51.57
HR_Th_	[min^–1^]	159 ± 14	153 ± 14	4.92	–25.70	35.54
$\dot{\text{V}}$O_2__Th_	[ml⋅min^–1^⋅kg^–1^]	32.2 ± 6.3	30.7 ± 3.6	0.64	–8.72	10.01
RER_Th_		0.96 ± 0.04	0.92 ± 0.05	0.03	–0.09	0.14

## Discussion

The main finding of the current study is that the GXT superior to determine peak physiological responses for elite athletes in handcycling. Both exercise tests and the corresponding lactate threshold concepts revealed appropriate estimates to predict a steady state performance for the majority of participants. However, as PO determination at LT_4 mmol L__–__1_ and LM exceeds the criteria for constant load performance (increase of ≥ 1 mmol L^–1^) for 33 and 17% respectively the current results indicate the common criteria for constant load performance (change of ± 1 mmolL^–1^) might not be sufficiently precise for elite athletes in handcycling. Consequently, exercise test results of elite athletes should be analyzed individually and verified by means of several CLT.

### Exercise Test Procedures – Peak Responses

Although the body of literature regarding physiological performance data of elite athletes in handcycling is limited peak values of $\dot{\text{V}}\,{\text{O}}_{2}$, RER, HR, La, and PO measured during both testing procedures in the present study are similar to previously reported results for highly trained paraplegic athletes in handcycling (Abel et al., 2006, 2010; Lovell et al., 2012; De Groot et al., 2014; Fischer et al., 2015) and wheelchair racing (Cooper, 1992).

While the GXT and the LMT are composed of different test protocols, obtained $\dot{\text{V}}\,{\text{O}}_{2}$ peak values were similar and significantly correlated (Table 2). Considering the range of dispersion both exercise test procedures can be applied to determine maximal aerobic capacity for elite athletes in handcycling. However, related values for peak PO differ significantly between the procedures. This relation is highly relevant for deriving training loads. As the ramp test revealed the highest result for $\dot{\text{V}}\,{\text{O}}_{2}$ and PO this exercise test might be the best choice in order to determine maximal aerobic capacity and to derive corresponding training loads as percentages of $\dot{\text{V}}\,{\text{O}}_{2}$ peak. Based on the nature of the test protocol the ramp test is generally applied to examine maximal cardiorespiratory responses (Hollmann et al., 2012). Current results for RER peak values confirm this application for handcycling. Since the highest HR response was obtained within the GXT, HR control might be regulated differently compared to able-bodied endurance athletes. Bland–Altman plots also display a considerable range of peak HR responses (Figure 2). A possible explanation could lie in the SCI that may result in a delayed systemic adaptation to increased physiological demands (Krassioukov and West, 2014). However, this was considered and compensated in a prolonged warm-up phase. Since workload increase in the ramp test of the LMT protocol is more rapid compared to the GXT protocol (20 W/min vs. 20 W/5 min) time to exhaustion is significantly lower for the ramp test (11.4 ± 1.5 vs. 48.3 ± 7.3 min). Thus, a more likely explanation might be that the significantly shorter duration of the ramp protocol in the LMT in combination with the steep workload increase is limited locally through an acidosis in the working muscle groups and performance during the prolonged GXT is limited centrally by the cardiorespiratory system (Bhambhani, 2002). Moreover, thermoregulation might also play a vital role as the heart is under greater strain during prolonged exercise in order to maintain a normal body temperature compared to a shorter exercise (Hopman et al., 1993a; Theisen et al., 2001b). Regarding the specific responses of HR, the GXT appears to be the best exercise test for elite athletes in handcycling to determine peak physiological responses.

### Threshold Concepts

The PO corresponding to a defined metabolic situation represents a more robust parameter for performance prediction and training description than $\dot{\text{V}}\,{\text{O}}_{2}$max in able-bodied athletes (Coyle et al., 1988; Jones and Rikli, 2000; Fahs et al., 2014). GXT and LMT are established efficient exercise testing procedures to predict a constant load performance. In the current study, both testing procedures revealed a similar PO at LM and LT_4 mmol L__–__1_ for elite athletes in handcycling. While mean values indicate a high variation for PO, individual data show a similar relation between both testing procedures as all but one participants revealed a higher PO at LT_4 mmol L__–__1_ (Figure 1) with a mean difference of 12 W. Compared to the other athletes this participant showed a comparatively early and very steep increase in La during the GXT resulting in a premature exercise test cessation and therefore a lower PO at the predicted LT_4 mmol L__–__1_.

Although all participants were highly trained paraplegic handcyclists classified in either H3 or H4 and ASIA category B individual SCI related characteristics lead to variable physiological limitations and performance responses. Possible reasons could lie in both, the neurologic lesion level and the autonomic completeness of the injury. A complete sympathetic pathway interruption results in a loss of central and reflexive cardiovascular control that limits maximal heart rate and impairs blood pressure regulation and blood redistribution (Krassioukov and West, 2014). Thus, blood pooling in the lower limbs is intensified and compromises the redistribution of blood volume during exercise (Dela et al., 2003; Thijssen et al., 2009). A reduced venous return, stroke volume, and cardiac output are the consequences and present the central limited endurance performance of paraplegic athletes. The peripheral limitation of SCI athletes’ endurance performance results from the paraplegic leg muscles. Muscles below the SCI atrophy due to the lacking innervation and contraction (Hoffman, 1986). Based on the loss of muscle mass combined with a change in fiber type composition toward less oxygen consuming type II fibers, lower limb muscles might have a reduced capacity to uptake and oxidize lactate during the upper body exercise. Individuals with chronic, complete SCI showed a 35% reduction in lower limb muscle cross-sectional area and volume compared to untrained able-bodied individuals (Olive et al., 2003). Although paralyzed athletes have a similar lactate elimination during submaximal arm-cranking exercise as able-bodied athletes, during late exercise and early recovery elimination rates were higher for able-bodied athletes (Leicht and Perret, 2008). As slowed elimination rates in the early stage of recovery for paralyzed athletes might affect accuracy for LM, threshold determination results in an underestimation of MLSS. Thus, lactate concentration would increase earlier during the second test part (step test) due to the delayed lactate elimination representing a minor recovery. Similar to able-bodied endurance PO prediction for constant load performance by the LMT results in an underestimation of the actual MLSS (Heck et al., 1991). This finding is also confirmed for wheelchair racing showing a 8–9 bpm higher HR at the original MLSS (change of ≤ 1 mmol L^–1^) compared to the MLSS prediction derived from the LMT (Perret et al., 2012).

### Verification of Maximal Constant Load Performance

The averaged 12 W lower PO at LM is reflected by generally lower responses during the corresponding CLT. Since $\dot{\text{V}}\,{\text{O}}_{2}$ responses did not differ significantly between both CLT this parameter might not be sufficiently sensitive to differentiate between exercises at the two different PO (137 ± 18 vs. 149 ± 34 W). According to the results the higher PO during the CLT at LT_4 mmol L__–__1_ is achieved through significantly higher HR, RER and especially BLa responses indicating that highly trained paraplegic athletes increase their performance during a 30 min constant-load handcycling exercise by upregulated HR and elevated BLa production rather than an increase in $\dot{\text{V}}\,{\text{O}}_{2}$. However, these enhanced demands are not observed and reflected in athletes’ perceived exertion because RPE responses did not differ significantly between the CLT. Thus, RPE might be an inappropriate parameter to control training intensity. Bland–Altman plots displayed a high dispersion of RER at LT_4 mmol L__–__1_ and LM indicating different proportions of energy supplying substrates (Frayn, 1983). Significant higher RER values during the CLT at LT_4 mmol L__–__1_ might be a result of the highly significant increased BLa values as $\dot{\text{V}}\,{\text{CO}}_{2}$ output is increased via the chemoreceptors in order to support hydrogen ion buffering (Wasserman et al., 1990). There is evidence for a negative correlation between levels of MLSS and the estimated masses of primarily engaged muscles when MLSS is measured for different exercise modalities. As a consequence both the PO per unit muscle mass and BLa at a given workload decrease with increasing mass of the primarily involved muscles (Kjaer et al., 1991; Beneke and Von Duvillard, 1996; Hoffman et al., 1996; Beneke et al., 2001). Since the muscle mass involved in arm cranking is lower than during cycling it seems likely that PO at LT_4 mmol L__–__1_ and LM appears at a higher absolute BLa combined with a lower PO per unit mass of working muscle (Beneke, 2003a). Although the majority of examined athletes showed a lactate steady state during both CLT, the PO associated with LT_4 mmol L__–__1_ and especially LM might not represent the maximal capacity for a constant load performance. Taking account of a higher risk of failure (67%) all measured parameters during the CLT at LT_4 mmol L__–__1_ are higher but still reach a steady state. Thus, PO prediction for constant load performance by GXT might be more precise. However, at least one additional CLT with a 5% higher PO would have been required to verify the constant load performance and a recommendation for an international standard procedure for exercise testing in handcycling cannot be concluded.

The application of the results is limited to athletes in handcycling of H3 and H4 classification. While a considerable heterogeneity was already given within a very close range of lesion levels, the influence of impaired sympathetic function involving multiply additional limitations increases with the injury level of the spinal cord and results again in altered physiological responses and adaptations to exercise.

## Conclusion

The main finding of the current study is that the GXT superior to determine peak physiological responses for elite athletes in handcycling. Both exercise tests and the corresponding lactate threshold concept revealed appropriate estimates to predict a steady state performance for the majority of participants. However, as PO determination at LT_4 mmol L__–__1_ and LM exceeds the criteria for constant load performance (increase of ≥1 mmol L^–1^) for 33 and 17% respectively the current results indicate the common criteria for constant load performance (change of ± 1 mmol L^–1^) might not be sufficiently precise for elite athletes in handcycling. Consequently, exercise test results of elite athletes should be analyzed individually and verified by means of several CLT.

## Ethics Statement

This study was carried out in accordance with the recommendations of the Declaration of Helsinki, the German Sport University Ethics Advisory Committee with written informed consent from all subjects. The protocol was approved by the German Sport University Ethics Advisory Committee.

## Author Contributions

TA, CP, SZ, and HS conceived and designed the experiments. SZ, OQ, and CS performed the experiments. CS, OQ, TA, CP, and HS analyzed and interpreted the data. CS, SZ, TA, CP, OQ, and HS prepared the manuscript. All authors read and approved the final manuscript and agreed to be accountable for all aspects of the work in ensuring that questions related to the accuracy or integrity of any part of the work are appropriately investigated and resolved.

## Conflict of Interest Statement

The authors declare that the research was conducted in the absence of any commercial or financial relationships that could be construed as a potential conflict of interest.
